# Driving Performance and Technology Acceptance Evaluation in Real Traffic of a Smartphone-Based Driver Assistance System

**DOI:** 10.3390/ijerph17197098

**Published:** 2020-09-28

**Authors:** Gheorghe-Daniel Voinea, Cristian Cezar Postelnicu, Mihai Duguleana, Gheorghe-Leonte Mogan, Radu Socianu

**Affiliations:** 1 Department of Automotive and Transport Engineering, Transilvania University of Brasov, 500036 Brasov, Romania; cristian-cezar.postelnicu@unitbv.ro (C.C.P.); mihai.duguleana@unitbv.ro (M.D.); mogan@unitbv.ro (G.-L.M.); 2 General Magic Technology, 500090 Brasov, Romania; rsocianu@generalmagic.com

**Keywords:** smartphone-based ADAS, user acceptance study, driver behavior, road safety, driving performance

## Abstract

Technological advances are changing every aspect of our lives, from the way we work, to how we learn and communicate. Advanced driver assistance systems (ADAS) have seen an increased interest due to the potential of ensuring a safer environment for all road users. This study investigates the use of a smartphone-based ADAS in terms of driving performance and driver acceptance, with the aim of improving road safety. The mobile application uses both cameras of a smartphone to monitor the traffic scene and the driver’s head orientation, and offers an intuitive user interface that can display information in a standard mode or in augmented reality (AR). A real traffic experiment consisting of two driving conditions (a baseline scenario and an ADAS scenario), was conducted in Brasov, Romania. Objective and subjective data were recorded from twenty-four participants with a valid driver’s license. Results showed that the use of the ADAS influences the driving performance, as most of them adopted an increased time headway and lower mean speeds. The technology acceptance model (TAM) questionnaire was used to assess the users’ acceptance of the proposed driver assistance system. The results showed significant interrelations between acceptance factors, while the hierarchical regression analysis indicates that the variance of behavioral intention (BI) can be predicted by attitude toward behavior.

## 1. Introduction

Traffic accidents caused 51 road deaths per million inhabitants in the European Union (EU) in 2019 [1]. The situation is worse in Eastern Europe, where more than 80 people per million inhabitants are losing their lives in road accidents each year (e.g., in Romania it’s 96 deaths/million). The number of deaths continues to rise worldwide, as road traffic injuries become the leading cause of death for children and young people aged 5–29 years [2]. The road safety challenge needs to be addressed as the number of vehicles increases every year. The automotive industry and researchers raise the safety standards with each new generation of vehicles, aiming to reduce the loss of lives in case of crashes. Complex integrated technologies can identify and interpret the surrounding environment and, in some situations, even react without the driver’s intervention. There are three technologies that have been proven to positively influence the drivers’ behavior: collision warning systems (CWS), pedestrian collision warning (PCW), and lane departure warning (LDW) [3,4]. The common belief is that, in time, because the majority of cars will be equipped with these features, the number of traffic crashes will be significantly reduced. However, smartphone-based ADAS already have the potential to increase traffic safety and represent a feasible solution for underdeveloped countries, where the adoption of new technology takes more time, due to the economic, social, and psychological differences.

### 1.1. Driver Assistance Systems

Collision warning systems which alert drivers when a dangerous situation is detected have the potential to reduce rear-end striking crashes by 27%. Furthermore, CWS with autonomous emergency braking (AEB) could potentially prevent or mitigate a frontal collision by up to 70% [5].

In response to collision warnings, the driver decelerates, and this can over time diminish the number of accidents [6,7,8]. On-road and simulator studies have demonstrated the potential of CWS [9,10]. The authors in [11,12,13] have shown that there was a decrease of the driver’s response time in simulated imminent collisions with warnings, in comparison with the same scenario without warnings. The ability to assess and maintain a safe headway (also known as following distance) can be obtained and improved by using a CWS, according to an on-road study [14]. The use of an application with CWS and LDW has been found to lead to a decrease of 45% in insurance claims [3], as well as influence drivers to adopt a defensive driving style [15].

After an investigation of 1070 crashes, the authors in [16] concluded that the number of accidents could have been reduced by 6.1% if the vehicles were equipped with LDW technology. An in-vehicle smart driving aid that provided safety and fuel-efficient driving advice in real-time was found to have a positive impact, such as increasing the headway to 2.3 s, and a 4.1% improvement in fuel consumption [17]. The findings of a large-scale European field operational test (FOT) revealed that the use of adaptive cruise control (ACC) combined with forward collision warning (FCW) shows positive effects on traffic safety (due to an increased headway, reduction of harsh braking) and fuel consumption [4]. In order to evaluate the experience of driving a car equipped with ACC and FCW, 215 subjects participated in a study [18]. Most of them believed that the safety systems had helped to prevent a crash and showed interest in having this type of technology on their future vehicle. Moreover, the National Transportation Safety Board (NTSB) recommends CWS to become standard for all vehicles [19,20].

Approximately 21% of all road fatalities in the EU in 2018 are represented by pedestrians, while Romania had the highest rate of 35 per million inhabitants [21], making pedestrian detection and avoidance systems a priority. Research in the field of vision-based automatic pedestrian detection has become consolidated due to the many innovations that were developed by scholars and the automotive industry. The wavelet transform [22], fuzzy classification [23], pyramid binary features [24], probabilistic templates [25], Adaboost, histogram of oriented gradients (HOG), and local binary pattern (LBP) features [26] are some of the most used algorithms in detecting, classifying, and tracking pedestrians.

Mobile driver assistance applications can be useful in changing the driver’s behavior by promoting a defensive driving and reducing driver inattention [27,28]. In-vehicle portable safety devices do not offer the precision of built-in safety functions; however, they come with the advantage of low cost, huge adoption potential and ease of use. Also, portable devices offer the possibility to assess new ADAS functionalities before implementing them as built-in safety functions in vehicles [29]. According to the same authors, high costs are the main reason for the low acceptance and adoption of CWS.

AR warnings offer promising means of increasing the driver’s safety, with the potential to reduce response time and improve road hazard detection [30,31]. In general, visual warnings are used for informative purposes, while auditory warnings indicate dangerous situations (object collision or lane departure). Smartphone-based ADASs usually use image processing to detect possible front collisions or driver inattention [32]. A mobile driver assistance application that has the capability to simultaneously process video stream from the front and back camera of a smartphone can give less false alerts, thus reducing the chances of becoming annoying. Visual warnings are always highlighting potential risky situations, while auditory alerts are only used when an imminent collision is detected, or when the driver is inattentive to the traffic scene. Authors in [33] found that hearing a beep alert reduced the frequency of crashes in impending dangerous situations.

Mobile applications that promote road safety are already commercially available for Android and iOS platforms. CarSafe [34] and iOnRoad [35] use camera switching to combine information regarding the traffic scene and the drivers’ behavior. However, the transition of data acquisition between the main camera and the secondary camera could lead to dangerous situations by missing video frames in some unpredictable situations, such as a runner crossing the road.

The use of an ADAS to monitor and asses the driver’s behavior in real-time presents great potential in increasing traffic safety [36,37]. Drivers’ inattention was found to be one of the main causes of crashes and recently has been associated with the use of a smartphone while driving [38]. Texting and reading are the main in-car activities that significantly degrade driving performance [39,40]. A recent study showed that handheld texting tasks can lead to a delayed response to sudden braking events, which increases the probability of rear end accidents by at least 2.41 times. Another key finding was that speech-based texting tasks had no impact on the response time [41]. A feasible solution to reduce cell phone use while driving could be to adapt safety trainings, as shown in a paper which investigated the behavior of truck drivers [42]. Another research indicated that there is a need to raise awareness, modify attitudes as well as increase the risk perception associated with the use of cell phone while driving / riding a motorcycle. Moreover, they suggested that this can be achieved thorough educational tools and targeted road safety campaigns [43]. One method to promote safe driving behaviors implies monitoring and rewarding drivers [44]. Reward-based interventions were proven to be effective in various areas of risk prevention [45]. However, some researchers argue that drivers should become aware of the benefits offered by ADAS and use them daily without receiving incentives [46].

**Figure 1:**
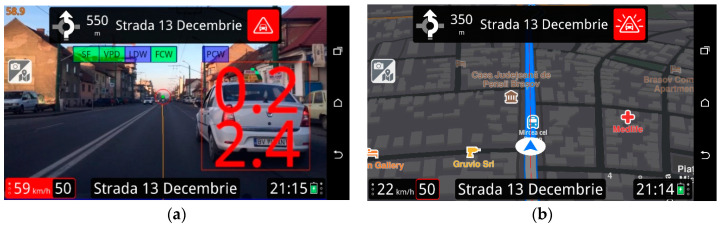
(**a**) Screenshot from the advanced driver assistance system (ADAS) application—default user interface using augmented reality; (**b**) Screenshot from the ADAS application—global positioning system (GPS) navigation user interface.

As inferred from previous studies, one of the main features that have deep implications on the acceptance of an ADAS is its user interface (UI). Consequently, we proposed an ADAS with an UI which is intuitive and flexible, as the user can choose between two types of interfaces (see Figure 1).

### 1.2. GPS Navigation Systems for Vehicles

Car navigation systems are widely used as standalone devices or by means of a smartphone. The aim of navigation systems is to guide the driver with turn-by-turn information to reach a destination. As such, this technology is mostly used when the route to a certain known destination is unfamiliar. There are several other advantages that come with recent smartphone GPS applications, like choosing an optimized route which is based on real-time traffic, speed limit warnings or finding nearby points of interest (e.g., gas stations, restaurants) [47]. The use of navigation systems is mainly influenced by the user interface and display quality components. The psychological factors that have an impact on the driver’s intention to use navigation systems are attitude and perceived usefulness, according to several studies that extend the TAM [48,49,50,51].

The impact and usage of navigation systems was also studied in a three-month FOT with 99 drivers. Results showed that there was no increase of critical driving situations because drivers adopted a safe behavior during system inputs (decreased mean speed, increased headway) [52,53]. Given the benefits and the specificity of this type of systems, our proposed assistance system offers the possibility to use a GPS-like UI.

### 1.3. Technology Acceptance Models of Driver Assistance Systems

Driver assistance systems have the potential to improve drivers’ performance and increase traffic safety; however, drivers can be reluctant to using them. The use of new technologies is mainly influenced by their acceptance and can be a sensitive and complicated topic, as it depends on many psychological and practical factors [54]. Researchers have studied driver acceptance of new technologies by applying several theories of human behavior and technology acceptance. First of all, driver acceptance of ADAS can be influenced by the drivers’ reaction when they are introduced to a new in-vehicle function or device, and their interest in adopting the technology while driving [55]. Authors in [56,57] analyzed how user experience (UX) can be integrated in the development of an ADAS, especially regarding the emotional reactions of the drivers using the system. The following factors were found to play a major role in evaluating products: usability, emotions (e.g., physiological reactions, behavioral tendencies) and aesthetic aspects. A novel structural model to assess the willingness to uptake smartphone driver support systems (SDSS) was presented in [58]. It aimed to examine the BI of young drivers by analyzing four variables—usability, social influence, perceived accuracy, and attitude. The model is based on previous well-known models, including TAM [59] and the unified theory of acceptance and use of technology (UTAUT) [60].

Three theoretical models of technology acceptance were identified to provide the necessary framework to define, characterize and estimate drivers’ acceptance: TAM, Theory of Planned Behavior (TPB) [61] and the UTAUT.

The TAM questionnaire was chosen to assess the drivers’ acceptance of the smartphone-based ADAS, as it provides more actionable information and brings more variance in BI [52] than the other 2 models. The actual usage of ADAS can be predicted with the help of three variables from the TAM questionnaire: perceived ease of use (PEoU), perceived usefulness (PU) and attitude toward behavior (ATT). The correlations between the TAM components offer a better understanding regarding the main features of a product that influences users. Thus, the study of drivers’ acceptance is crucial and should be addressed in the early stages of development and implementation. A recent model of drivers’ acceptance based on five components (attitude, perceived usefulness, endorsement, affordability, and compatibility) was able to predict 85% of the variability in drivers’ acceptance of driver support systems [62]. More research concerning the drivers’ acceptance of assistive technologies is essential in the context of our changing society, where technology is becoming more accessible and ubiquitous.

Reliable warnings generated quicker brake response as the driver is not slowed down by a visual search of hazardous situations [63]. An important factor that needs to be addressed is how to create a trustworthy relationship between the driver and the system [64]. False alarms can diminish drivers’ acceptance of ADAS and have the potential to create annoyance [65]. Other behavioral effects of annoyance include slower braking responses or even the tendency to ignore and turn off warnings [66]. If a turn-off switch for FCW crash alerts were available, about 41% of the subjects stated that they would use it because of the excessive and/or recurring false alerts [67].

### 1.4. Objectives of the Study

While navigation systems are widely known and used, mobile ADAS applications are not as popular and drivers appear to be reluctant in accepting them [44,68]. In this study we present a viable solution that could increase the acceptance of driver assistance systems by combining a navigation application with collision warning features. The proposed mobile application is an ADAS that offers turn-by-turn navigation information, while monitoring the traffic scene and the driver. By analyzing the video stream from both cameras of a smartphone, we aimed to create a low-annoyance mobile driver assistance system which issues alerts only when the driver is inattentive. The application was tested by 24 participants in a real driving experiment.

## 2. Materials and Methods

### 2.1. Participants

Twenty-four participants (20 males and 4 females; mean age = 29.38 years, SD = 6.70 years) were recruited from the student and professor body of the Transilvania University of Brasov, Romania. The only prerequisite for taking part in the experiment was the possession of a valid driver’s license. The participants had a license for an average period of 9.71 years (SD = 5.77 years) and estimated to drive on average 12,917 km per year (SD = 3604.55 km). They have used mobile GPS navigation applications at least once; 65% admitted that they use them at least once a month. Regarding the use technologies that can detect dangerous situations (such as vehicle collision, pedestrian collision, or lane departure), only six had previous experience.

### 2.2. Tools and Instruments

The vehicle used in the study was a Skoda Octavia, without integrated navigation software or other collision detection features. The driver assistance system was installed on an HTC (HTC Corporation, New Taipei City, Taiwan) One M9 smartphone with the following characteristics: Qualcomm Snapdragon 810 processor at 2 GHz, Adreno 430 GPU, 3 GB RAM, a primary camera of 20 MP, f/2.2, 28mm, a secondary camera of 4 MP, f/2.0, 27mm. A second HTC One M9 was used to video record the entire driving experiment (simultaneously recording the driver and the traffic scene) for the purpose of verifying all the issued warnings and alerts. This device was chosen because, at the moment of the study, it was among the few commercial smartphones that could allow Android developers to simultaneously access the front and back camera video streams. The main camera of the smartphone is used for real-time monitoring of the traffic scene, while the front-facing camera is used for monitoring the drivers’ head orientation. The video stream from the back camera is processed by a lane departure module, as well as vehicle and pedestrian collision modules.

The output from the aforementioned modules consists of 3 warnings and 3 alerts that are presented to the user in a low annoyance manner, based on the driver’s head orientation. The concept is to present, initially, only visual stimuli to inform the user about warnings, while auditory stimuli will be issued if the driver is not paying attention to the traffic scene. In comparison, alerts were defined as the simultaneous visual and auditory warnings that are issued when an imminent collision is detected, regardless of the driver’s head orientation. This approach has the advantage of reducing the number of messages that are delivered to the driver. The auditory stimulus was composed of two sound bursts, each burst containing a continuous stimulus of 0.6 s at a frequency of 2500 Hz, and which last approximately 1.31 s [69]. Bursts were separated by 110 ms and had an amplitude of 70 dB without an envelope [32]. The visual warnings for collisions consisted of a flashing red triangle, while for lane departure a distinct representative visual icon was used. By using different icons, the users can understand the reason for receiving specific warnings and this was proven to increase confidence in the system [62].

The warnings and alerts that can be issued by the proposed application are the following:

- Lane departure left/right solid warnings: issued when the vehicle crosses the solid line.
- Nearby vehicle warning: issued at a speed of less than 30 km/h, when the distance to the front vehicle is less than 0.6 m (distance until collision).
- Dangerous headway alert: when time to collision (TTC) is less than 0.8 s.
- Vehicle collision alert: issued when TTC is 2.7 s, which is enough time for the driver to react and avoid an accident.
- Pedestrian collision alert: issued when TTC with a pedestrian is less than 3 s.

The smartphone is placed on the windshield at a location that allows the front camera to record the driver’s face and the back camera to see the traffic ahead, according to the calibration procedure presented by [70]. When the smartphone is positioned horizontally (landscape orientation), the collision warning modules are automatically activated. A detector based on LBP codes and the Adaboost cascade classifier is used to identify possible obstacles (cars, trucks, pedestrians, etc.), then each detected obstacle is validated by a classifier, based on HOG and neural networks. If the result is positive, a Kalman filter updated recursively is used to track the obstacle.

The recognition step that follows the detection is where all or almost all false detections are eliminated. The detected obstacles are categorized based on the properties described with HOG. The classifier was trained by using a database of textures, containing about 30,000 positive textures and a similar number of negative textures, which were manually selected from an area of interest where is the highest probability to find obstacles. The accuracy for the recognition of pedestrians was of 98%, 95% for vehicles and 90% for trucks (during daytime, real traffic experiments). A thorough description of the object detection and recognition cannot be provided as the intellectual property is shared with a private company.

According to [67] most assistance systems only require the coarse gaze direction. Thus, the developed application for this study uses the Euler Z and Y angles of the drivers’ head to determine its orientation. The application allows a real-time measurement of the drivers’ head direction at a rate of 25 Hz (maximum 25 frames per second), identifying if the driver is paying attention to the traffic scene or not.

### 2.3. Study Variables

The demographic and driving performance variables analyzed in the present study are presented in Table 1. The proposed ADAS application in this study has a TTC threshold set to 0.8 s which triggers a dangerous headway alert. The value was chosen based on the relevant literature review. The authors in [71] conducted a long-term study regarding the impact of a following distance warning system. The first level of warning was issued at a TTC of 1.7 s and it continues to visually inform the driver until it reaches a TTC of 1.0 s when the color of the visual icon changes from yellow to red, and then at a TTC of 0.8 s triggers an auditory and visual warning. They also conclude that a large-scale deployment of collision warnings system will not occur until the nuisance and false warnings generated by the application are minimized, thereby increasing driver acceptance. A 1.1 s TTC at an average speed of 50 km/h was used in [72]. In studies that do not monitor the driver’s attention, a higher TTC is generally used to compensate for the potential inattention of the driver.

### 2.4. Study Procedure

**Figure 2:**
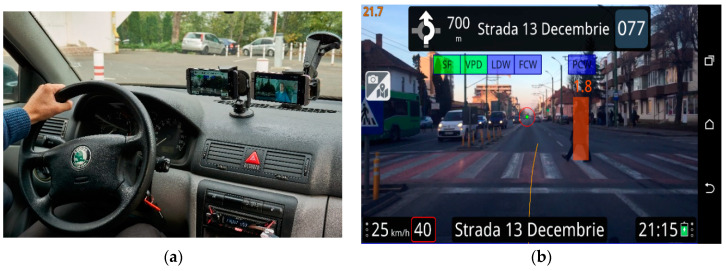
(**a**) The setup for the experiment, with the two smartphones positioned horizontally (one with the ADAS, the second for experiment video recording); (**b**) Screenshot from the ADAS application—pedestrian detected.

A real driving experiment was conducted in Brasov, Romania. The chosen route is 10 km long and was estimated to last around 20 min to complete, with moderate traffic. The driving path has roundabouts, traffic lights, pedestrian crossings and up to four lanes. The setup of the experiment presenting the position of the smartphones can be observed in Figure 2a.

In order to comply with the Directive 1995/46/EC regarding the protection of personal data and with the Directive 2002/58/EC regarding the privacy, participants have been informed about the scope of the experiment and were given a consent agreement form to sign.

The visual warnings for vehicle collision, pedestrian collision and solid line crossing warnings have been presented to the participants, as well as the auditory stimulus (a screenshot from the ADAS application showing a visual warning of a pedestrian collision is presented in Figure 2b).

Having undergone a 5–10 min. practice session, each participant became familiar with the car and the navigation assistant. Before starting the test, an observer instructed each participant to follow the turn-by-turn information given by the proposed driver assistance application that guided them through the experiment.

The experiment consisted of two separate phases. Firstly, the participants used the application to navigate to the destination and received no feedback/warnings/alerts (Baseline scenario). Secondly, the participants followed the application’s directions and received visual and auditory feedback whenever a dangerous situation was detected (ADAS scenario). This sequence of testing was not varied and the no-ADAS scenario was always first. In both scenarios, the application was in the default setting that superimposes information on top of the live video stream from the back camera of the smartphone. When participants reached the destination, they were de-briefed and asked to complete the TAM questionnaire to assess the acceptance of the proposed ADAS mobile application.

Traffic congestion is a common problem in modern cities as the number of cars is rising exponentially, while progress in infrastructure doesn’t always respond to real situations. The experiment took place outside rush hours, between 9:00 a.m. and 1:00 p.m. Driving during the selected time interval had the advantage of a low to moderate traffic. In order to validate the two scenarios, the observer had the option to cancel the experiment if its duration exceeded 35 min due to intensive traffic, therefore ensuring similar road conditions for all participants.

### 2.5. Technology Acceptance Model and Hypothesis

**Figure 3:**
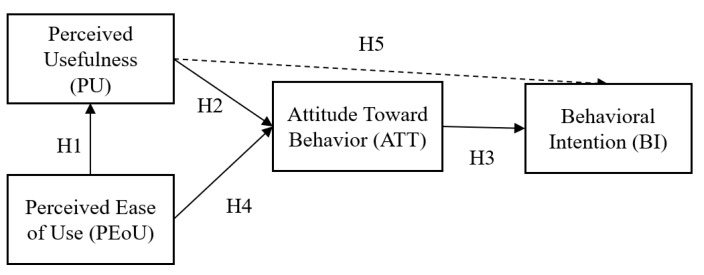
Representation of the proposed technology acceptance modelbased hypothesis.

The technology acceptance is evaluated by means of a 17-item acceptance questionnaire (see Appendix A) which analyses three components of BI: ATT, PU and PEoU (see Figure 3). According to TAM, positive ATT and high PU and PEoU reveal a high intention to use a technology. Attitude is considered to be the emotional state that a person has towards using a technology [74]. Perceived usefulness reveals the degree to which a person thinks that his or her performance could be improved by using a specific system [59]. The degree of difficulty in using a certain technology is evaluated by PeoU.

Understanding which factors influence the drivers’ acceptance is crucial and should be addressed when designing driver assistance systems. The following hypotheses are proposed (see Figure 3):

**Hypothesis** **(H1).**  *Perceived ease of use has a positive significant effect on perceived usefulness.*

**Hypothesis** **(H2).**  *Perceived usefulness has a positive significant effect on attitude toward behavior.*

**Hypothesis** **(H3).**  *Attitude toward behavior has a positive significant effect on behavioral intention.*

**Hypothesis** **(H4).**  *Perceived ease of use has a positive significant effect on attitude toward behavior.*

**Hypothesis** **(H5).**  *Perceived usefulness has a positive significant effect on behavioral intention.*

## 3. Results

The data collected during both Baseline and ADAS scenarios were saved in.csv files and further processed using SPSS (IBM, New York, USA). The number of issued warnings and alerts was verified by visually analyzing the video recordings of the experiments for each participant. Paired *t*-tests analysis was conducted to find relevant statistical differences between the baseline and ADAS scenarios.

### 3.1. Driving Performance Assessment

**Figure 4:**
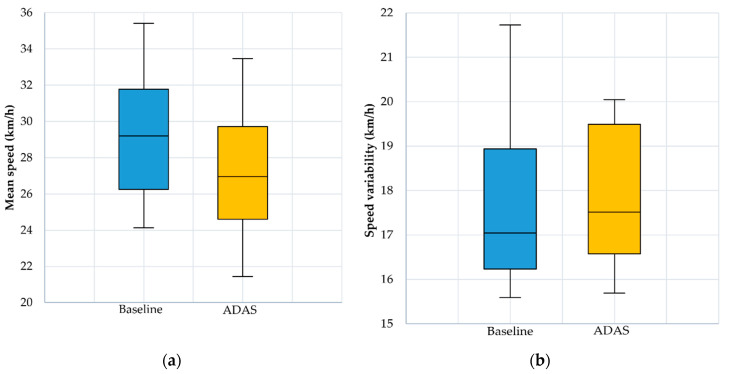
(**a**) Mean speed boxplots; (**b**) Speed variability boxplots.

**Figure 5:**
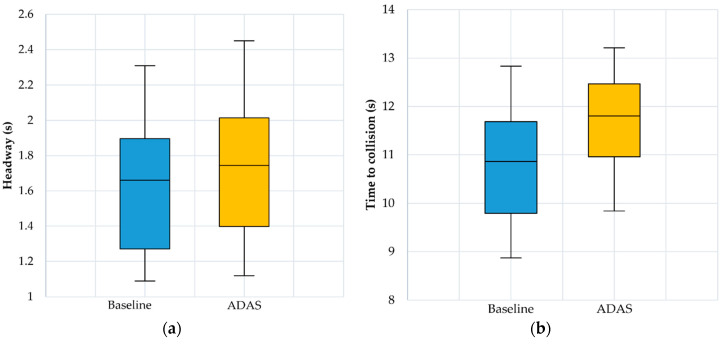
(**a**) Headway boxplots; (**b**) Time to collision boxplots.

**Figure 6:**
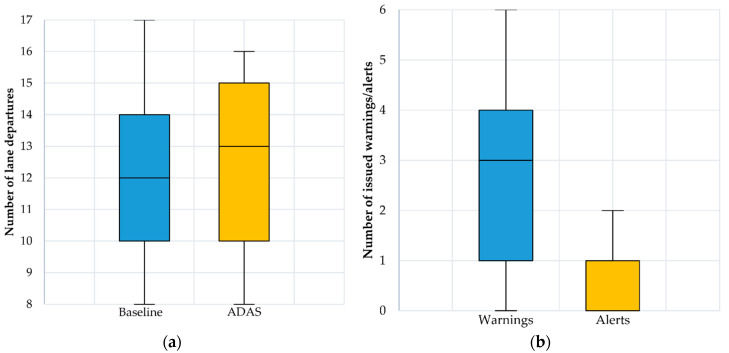
(**a**) Number of lane departures boxplots; (**b**) Numbers of warnings and alerts obtained during the ADAS scenario.

The results for mean speed and speed variability are shown in Figure 4. The results for headway and TTC are presented in Figure 5, while the number of lane departures obtained for both scenarios and numbers of warnings and alerts obtained during ADAS scenario are presented in Figure 6.

By running the paired samples *t*-test for the Baseline and the ADAS scenarios, statistical significant differences were obtained for the mean speed parameter: t (23) = 9.385, *p* < 0.05 (Baseline speed: 29.097 ± 3.516 km/h, ADAS speed: 27.219 ± 3.324 km/h), headway t (23) = −7.281, *p* < 0.05 (Baseline headway: 1.638 ± 0.372 s, ADAS headway: 1.742 ± 0.395 s) and TTC t (23) = −6.213, *p* < 0.05 (Baseline TTC: 10.781 ± 1.147 s, ADAS TTC: 11.67 ± 0.963 s) parameters, while for the number of lane departures t (23) = −0.811, *p* = 0.426 (Baseline lane departures: 12.042 ± 2.458, ADAS lane departures: 12.292 ± 2.789) there were no statistical significant differences.

The average number of issued warnings was of 2.63 (SD = 1.61) and the average number of alerts was of 0.58 (SD = 0.72).

### 3.2. TAM Results

#### 3.2.1. Data Processing and Analysis

The data from the TAM questionnaire were centralized by using Excel, while SPSS was used for the statistical analysis. All items were measured on a 7-point Likert scale, where the points represent the following: 1 = strongly disagree, 2 = moderately disagree, 3 = somewhat disagree, 4 = neutral (neither disagree nor agree), 5 = somewhat agree, 6 = moderately agree, and 7 = strongly agree. For the reverse-scaled constructs the scores were subtracted from 8 to keep the consistency of the scale. For the statistical analysis, the average of the participants’ ratings was determined for the survey items under each construct. A hierarchical regression analysis was used to explain the variance in behavioral intention or, in the present study, the drivers’ acceptance of the proposed system.

#### 3.2.2. Reliability of Scales and Descriptive Statistics

The descriptive statistics are presented in Table 2. The means of the four constructs range from 4.83 to 5.90 and their standard deviation range from 0.58 to 0.89.

#### 3.2.3. Hierarchical Linear Regression Analysis

The hypotheses of this study have been tested by using the hierarchical linear regression analysis. Three models that use PU, ATT and BI as dependent variables are defined as follows:

Model 1, with PU as the dependent variable, determines the validity of H1, i.e., the influence of PEoU on PU.

Model 2 has ATT as dependent variable and it aims to examine the influence of PEoU and PU over ATT, i.e., H2 and H4.

Model 3 with BI as the dependent variable aims to evaluate the impact of two independent variables, namely PU and ATT (H3, H5) over BI. The results are numerically presented in Table 2, Table 3 and Table 4 and as structural links of the proposed model in Figure 5.

##### Model 1 (Perceived Usefulness)

As shown in Table 3, the construct PEoU has a significant positive effect on PU (β = 0.44, *p* < 0.05). This indicates that high ratings on PEoU will lead to a positive PU, thus supporting H1.

##### Model 2 (Attitude toward Behavior)

The results of Model 2 are presented in Table 4. In Step 1, PEoU is inserted into the hierarchical regression model. In Step 2, PU is added subsequently to the regression model. PU increases the variance of ATT by a significant 34% (R^2^ = 0.16, increasing to 0.50 in the second step), thus H2 is supported. Among the two independent variables that can predict ATT, PU has the largest impact in terms of regression coefficients (β = 0.64, *p* < 0.01). PEoU does not have a significant effect on ATT, thus concluding that H4 is not supported.

##### Model 3 (Behavioral Intention)

The results of Model 3 are shown in Table 5. Three independent variables represent the input data for the model: PEoU, PU and ATT. The obtained results suggest that the addition of ATT improves the variance in BI significantly (ΔR^2^ = 0.34, *p* < 0.05). The regression coefficient of PU is not significant, thus revealing that H5 is not supported. PEoU was found to have a relatively strong relationship with BI (R^2^ = 0.31, β = 0.55). The hierarchical regression reveals that the ATT is the most powerful predictor toward usage intention for the developed application, which indicates that H3 is supported.

**Figure 7:**
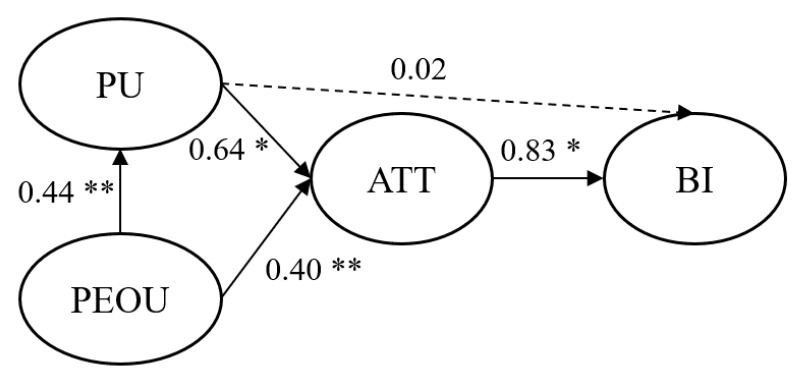
Estimated model of the study. *Notes:* Weights on arrows represent standardized regression estimates (βs). Dashed arrows indicate insignificant effects. * *p* < 0.01, ** *p* < 0.05.

The resulting model of the study is presented in Figure 7 The findings are discussed in the following chapter.

## 4. Discussion

### 4.1. Driving Performance

The findings clearly indicate that using an assistance system has a positive impact on the driver’s behavior. The most important effects represent the increase in time headway and a lower mean speed. Time headway is one of the main indicators which estimates the criticality of certain traffic circumstances. Small headways generate potentially dangerous situations, and in some countries, this indicator is used for law enforcement, as drivers can receive a ticket for close following [73].

Experiments that take place in real traffic conditions are more relevant than those which take place in a simulator, but they present some challenges and risks as well. The participants stated that a traffic jam can influence their willingness to use an ADAS because they usually find themselves using their phone for other distracting tasks, such as talking, gaming, or using a social network application. All participants reported that they were familiar to the chosen path and road condition in Brasov, therefore we hypothesized that their driving performance during the two scenarios would not be affected by learning effects. Several aspects were taken into consideration when working on the collision and lane departures modules. The total time required to stop the vehicle consists of the driver’s reaction time and the braking time necessary for the deceleration of the vehicle. The braking time (*t*) is determined by the driving speed and the braking deceleration (time = velocity/acceleration). The velocity refers to that of the vehicle equipped with the ADAS. The total distance covered before a vehicle stands still consists of the “reaction distance” (reaction time × velocity) and the braking distance (m) that is covered while braking. The braking distance is related to braking deceleration and braking time as follows:

S = ½ × a × *t*^2^ (1)

For example, considering a “reasonably quick” reaction of the driver (of 1 s: 0.5 s perception time + 0.2 s reaction time, required to release the acceleration and to press the brake pedal, +0.3 s for the driver’s unexpected behavior) in suboptimal circumstances, a constant braking deceleration of 5 m/s^2^ and 1 s reaction time on a wet road surface, in the case of an emergency stop at 80 km/h, the total braking time is far above 5 s and the total braking distance is about 70 m. In the case of an emergency stop at 120 km/h, the total braking time is of nearly 8 s and the total braking distance practically doubles, to more than 140 m. Braking distances are shorter on a dry road surface, but the disproportionally longer braking distance at higher speeds remains. Nevertheless, it is clear that even at fairly low speeds, more than 2 s are needed for a complete an emergency stop, even when there is a reasonably quick reaction time of 1 s. We emphasize therefore that a 2 s headway is only sufficient to make an emergency stop possible and keep a safe distance from the vehicle in front, when the car approaches gradually (with clear knowledge of the front vehicle existence and with a relatively low speed that ensures a smaller reaction time). When the car is travelling at higher speed, the 2 s rule is overruled as the situation becomes too dangerous.

Another relevant finding is an increase in TTC. Authors in [75] stated that a 3.6–5.6 s TTC interval corresponds to a critical time during which the driver must quickly respond and take action in order to avoid an accident. A collision course of two road users is a pre-requirement in order for a crash to happen. However, encounters without a collision course can have crash potential as well, since any minor change in the car’s direction can lead to a potentially dangerous situation. Therefore, TTC alone is not sufficient to increase traffic safety [76].

The mean speed was found to be statistically significant and slightly smaller in the ADAS scenario. No major differences were found regarding the number of lane departures between the two scenarios. This finding was to be expected because of the urban context in which the experiment took place and the same itinerary path used for the two testing scenarios.

Road safety has improved greatly in the past decades due to technological advances, regulations and policies that focus on the prevention of deaths and injuries. For example, many countries are pushing hard to include automatic emergency braking systems as a standard feature for all new cars that will be sold starting from 2022 [77].

While using an ADAS, the driver’s behavior can change and adapt to the new circumstances. In some cases, the use of a warning system generated negative behavioral adaptations like: a decrease of minimum time to collision, increase of maximum speed or even an increased intensity while performing a secondary task compared to driving without an ADAS [78]. The adapted behavior may limit the intended safety effect of the ADAS and it is advisable to have a comprehensive research regarding the positive and negative impacts that assistance systems have on the driver’s behavior. As such, the next step for validating the proposed mobile ADAS will be to evaluate the effects of a long-term use with a larger sample size.

The driver’s performance is influenced by external and more important, internal factors. While the traffic and weather cannot be changed, the driver’s state of mind can easily be affected by exhaustion, irritability, or anxiety. Therefore, assistance systems should be designed to be reliable, easy to use and without annoying the user. Providing information in the right moment, as opposed to flooding the driver with warnings and alerts, is highly recommended [79]. The proposed driver assistance application monitors the driver’s head orientation to reduce the number of warnings, thus increasing the user’s confidence in the application, which in turn can lead to long-term use.

### 4.2. User’s Acceptance of the Proposed Driver Assistance Application

The TAM questionnaire was used to examine the factors that influence the drivers’ acceptance of the proposed ADAS. The internal consistency for all scales was found to be high, with a range for Cronbach’s alpha (α) from 0.70 to 0.87 (a value higher than 0.60 indicates an acceptable consistency for the constructs) [80]. The inter-correlations among constructs were significant (see Table 2).

Results showed that drivers’ acceptance of in-vehicle assistance systems is influenced primarily by the attitude and perceived usefulness constructors. The hierarchical regression also revealed that PEoU has a statistical significance on predicting drivers’ behavioral intentions. The intention to use the proposed ADAS can be predicted by attitude, which is a sum of an individual’s positive or negative feelings (about performing the target behavior [74]). The significant effect of PU and ATT on BI is in line with the TAM literature [47,55,81]. The present study also found a larger effect for PU on ATT compared to PEoU. This result supports the hypothesis that ATT is formed based on relevant beliefs. Thus, a driver is inclined to use an ADAS if he finds it useful and easy to use [82].

The PEoU construct measures the user’s perception regarding the effort required to use the system. The user interface should offer a clear and understandable interaction, whilst not requiring complex technical knowledge. The low annoyance approach of the proposed ADAS, coupled with turn-by-turn navigation guidance and a user interface with augmented reality, has received high ratings for the PEoU construct. The results of Model 3 of the hierarchical regression showed that PEoU accounts for a significant 22% variance in BI.

When drivers receive auditory warnings (for collision avoidance) that were not useful, otherwise considered false, they are inclined to express fewer positive reactions [6]. A smartphone-based ADAS may be less accurate than the built-in safety features of high-end cars. Nonetheless, research in this area is needed in order to increase awareness and create reliable and user-friendly solutions. Overall, participants were not annoyed by the warnings and showed a positive reaction to the proposed ADAS.

Based on the findings of the current study and previous smartphone-based ADAS research, a mobile driver assistance application could help increase road safety by offering relevant and real-time information regarding the road environment. A key component of road safety is represented by driving styles, which are different for each individual and depend on the drivers’ personality and sociodemographic characteristics. A recent study on crossing collision prevention systems (CCPSs) concluded that there is a heterogeneity in the effectiveness of CCPS caused primarily by driving styles [83]. We believe that the heterogeneity among drivers in our study is caused primarily by their driving experience, which could make professional/experienced drivers more reluctant to use a smartphone-based ADAS. Driver assistance systems which alert drivers of possible collisions could be useful in preventing crashes that involve vehicles, pedestrians, as well as motorcyclists or cyclists. A recent study highlighted the lack of perception of motorcyclists and the relation between the motorcyclist’s characteristics and test context that influence the low detection of a vehicle’s turning signals [84].The integration of assistance features, such as collision warning and lane departure, in addition to the tracking of the driver’s head orientation, coupled with an already widely used and accepted technology such as GPS navigation and an intuitive AR user interface, can increase the acceptance of smartphone-based ADAS among drivers.

The presented study poses some limitations. The method to assess the driver’s inattention by only tracking head orientation could lead to false alerts. Therefore, future research should focus on detecting the driver’s eye gaze and/or fatigue to ensure a more reliable system and, as a result, could increase the driver’s confidence in the system. Another limitation is the small scale of the experiment, with a reduced number of participants and a short test duration of just twenty minutes. Future research should also investigate the influence of specific voice warnings instead of the auditory stimulus, which might enhance the human–computer interaction.

## 5. Conclusions

This study investigates the use of a smartphone-based ADAS for navigation and collision warning. The mobile application was tested in real traffic conditions with twenty-four participants. The addition of collision detection and driver’s head orientation tracking to a GPS navigation application generated a positive change in the driver’s behavior and led to the adoption of a more defensive driving style. A TAM questionnaire was used to gather subjective data which revealed that the main factors that influence the driver’s acceptance of the proposed application were attitude and the perceived usefulness. The results of this case study are promising and highlight the potential of a smartphone-based, low-cost, and low-annoyance ADAS in increasing road safety. As smartphones are becoming a part of our everyday life, we expect to use them in any situation. The number of smartphone users has almost doubled in the past five years and has reached 2.87 billion in 2020. This creates new possibilities to generate safer behaviors for drivers of vehicles without integrated collision or lane detection feature by simply installing a driver assistance application.

Road safety can be improved by educating drivers and raising awareness about the possible consequences that arise from not paying attention to the traffic scene. Driver assistance systems that monitor both the driver and the traffic scene pose great potential in decreasing the number of crashes by offering reliable and timely warnings. Because the ADAS would be installed on their device, this might reduce the possibility for drivers to use the smartphone for other purposes (e.g., social media, texting).

## Figures and Tables

**Table 1:** Study demographic and driving performance variables.

Category	Variable Name	Short Description
Demographic variables	Age	
	Gender	
	Driving experience (number of years)	
	Estimated annual mileage (km)	
Driving task performance:	Speed control.	Two variables were used to assess the speed control: (a) mean speed (km/h), calculated with V = (x1 − x0)/t, with x1 − x0 representing the length of the road and t is the time needed to complete the driving test; (b) speed variability, represented by the standard deviation of the driving speed (km/h).
	Time Headway	Time headway is an indicator of criticality for a given traffic scene and represents the time needed by the following vehicle to reach the same point as the lead vehicle [73].
	Time to Collision	TTC is defined as the time until a collision would happen if two successive vehicles keep their course and speed unchanged.
	Lane departure.	This variable was defined by the number of lane departures. A lane departure (left or right) was determined when the vehicle has crossed the driving lane boundaries.

**Table 2:** Internal consistency of the scales, correlations and descriptive statistics (*n* = 24).

Variable	Mean	SD	PEoU	PU	ATT	BI
PEoU	4.83	0.76	0.74			
PU	5.22	0.89	0.43 *	0.70		
ATT	5.71	0.58	0.40 *	0.70 *	0.87	
BI	5.90	0.69	0.55 *	0.69 *	0.93 *	0.75

Notes: * *p* < 0.01. PEoU—perceived ease of use. PU—perceived usefulness. ATT—attitude toward behavior. BI—behavioral intention.

**Table 3:** Hierarchical regression analysis on Perceived Usefulness (PU).

Independent Variable	Step 1
PEoU	0.44 **
R^2^	0.19 **
F-value	5.18 **

Note: Variable denotations see Figure 3. ** *p* < 0.05.

**Table 4:** Hierarchical regression analysis on attitude toward behavior (ATT).

Independent Variable	Step 1	Step 2
PEoU	0.40 **	0.12
PU		0.64 *
R^2^	0.16 **	0.50 *
Adjusted R^2^		0.46 *
F-value	4.37 **	10.61 *
ΔR^2^		0.34

Note: For variable denotations see Figure 3. * *p* < 0.01, ** *p* < 0.05.

**Table 5:** Hierarchical regression analysis on Behavioral Intention (BI).

Independent Variable	Step 1	Step 2	Step 3
PEoU	0.55 *	0.31	0.21 **
PU		0.56 *	0.02
ATT			0.83 *
R^2^	0.31 *	0.56 *	0.90 *
Adjusted R^2^		0.52	0.89
F-value	9.79 *	13.26 *	62.59 *
ΔR^2^		0.25	0.34

Note: For variable denotations see Figure 3. * *p* < 0.01, ** *p* < 0.05.

## Appendix A

**Table A1:** Survey questions and their relation to the acceptance measures.

Acceptance Measure	Survey Question	Scale
Attitude toward behavior	“The use of the system when I am driving would be”	Bad (1) −> Good (7)
	“The use of the system when I am driving would be”	Useless (1) −> Useful (7)
	“The use of the system when I am driving would be” *	Desirable (1) −> Undesirable (7)
	“The use of the system when I am driving would be”	Ineffective (1) −> Effective (7)
	“The use of the system when I am driving would be”	Sleep-inducing (1) −> Alerting (7)
	“The use of the system when I am driving would be”	Unpleasant (1) −> Pleasant (7)
	“The use of the system when I am driving would be”	Extremely annoying (1) −> Not at all annoying (7)
	“The use of the system when I am driving would be”	Irritating (1) −> Likeable (7)
	“The use of the system when I am driving would be” *	Assisting (1) −> Worthless (7)
Perceived Usefulness	“Using the system would improve my driving performance”	Strongly disagree (1) −> Strongly agree
	“Using the system in driving increases my safety”	Strongly disagree (1) −> Strongly agree
	“Using the system enhances effectiveness in my driving”	Strongly disagree (1) −> Strongly agree
	“I would find the system useful in my driving”	Strongly disagree (1) −> Strongly agree
Perceived Ease of Use	“My interaction with the system would be clear and understandable”	Strongly disagree (1) −> Strongly agree
	“I would find the system difficult to use” *	Strongly disagree (1) −> Strongly agree
	“Interacting with the system would not require a lot of mental effort”	Strongly disagree (1) −> Strongly agree
	“I would find it easy to get the system to do what I want it to do”	Strongly disagree (1) −> Strongly agree
Behavioral Intention	“If the system is available in the market at an affordable price, I intend to purchase the system”	Strongly disagree (1) −> Strongly agree
	“If my car is equipped with a similar system, I predict that I would use the system when driving.”	Strongly disagree (1) −> Strongly agree
	“Assuming that the system is available, I intend to use the system regularly when I am driving.”	Strongly disagree (1) −> Strongly agree

* Reverse-scaled item.
